# Shortening the sulfur cell cycle by a green approach for bio-production of extracellular metalloid-sulfide nanoparticles

**DOI:** 10.1038/s41598-023-31802-6

**Published:** 2023-03-23

**Authors:** Farnoush Asghari-Paskiabi, Mohammad Imani, Hashem Rafii-Tabar, Seyed Ali Nojoumi, Mehdi Razzaghi-Abyaneh

**Affiliations:** 1 Department of Medical Physics and Biomedical Engineering, School of Medicine, Shahid Beheshti University of Medical Sciences, Tehran, Iran; 2 Department of Mycology, Pasteur Institute of Iran, Tehran, 1316943551 Iran; 3 Novel Drug Delivery Systems Department, Iran Polymer and Petrochemical Institute, P.O. Box 14975-112, Tehran, Iran; 4 Institute for Nanoscience and Nanotechnology, Sharif University of Technology, Tehran, 14588-89694 Iran; 5 Department of Mycobacteriology and Pulmonary Research, Pasteur Institute of Iran, Tehran, Iran; 6 Microbiology Research Center (MRC), Pasteur Institute of Iran, Tehran, Iran

**Keywords:** Biotechnology, Microbiology, Molecular biology, Nanoscience and technology

## Abstract

In the present study, a new approach was introduced regarding the extracellular synthesis of selenium sulfide micro/nano-particles using *Saccharomyces cerevisiae* in different ammonium sulfate supplementation and in the presence of sodium selenosulfate precursors (S_1_) and a blend of selenous acid and sodium sulfite (S_2_). In S_1_, only cell supernatant exposed to ammonium sulfate was able to reduce sodium selenosulfate. Whereas, in S_2_, cell supernatant in both pre-conditions of with or without ammonium sulfate (S_2_ + or S_2_−) were able to reduce selenous acid and sodium sulfite. Electron microscopy, also indicated that selenium sulfide NPs were successfully synthesized with average size of 288 and 332 nm for S_2_ + and S_2_− in SEM and 268 and 305 nm in TEM. Additionally, elemental mapping by energy-dispersive x-ray analysis confirmed the presence of sulfur/selenium elements in the particles in a proportion of 24.50 and 23.31 for S_2_− and S_2_ + , respectively. The mass spectrometry indicated the probability of Se_2_S_2_, SeS_1.1_, Se_2_, Se, SeS_5_, SeS_3_, Se_3_S_5_/Se_5_, Se_3_/SeS_5_, Se_6_, Se_4_/SeS_7_, Se_2.57_S_5.43_/Se_2_S_2_ and Se_4_S/Se_2_S_6_ molecules for S_2_ + and of Se, Se_2_, Se_3_S_5_/Se_5_, Se_6_ and Se_4_ species for S_2_−. In FTIR spectra, primary (i.e. 1090–1020 and 1650–1580 cm^−1^) and secondary (1580–1490 cm^−1^) amine bands duly confirmed the protein corona around the NPs.

## Introduction

Selenium sulfide is mostly applied to cure folliculitis, pityriasis versicolor, dermatitis^1^ and hyperkeratosis^2^. Polydispersed selenium NPs, synthesized through aqueous leaf extract of lemon plant, were, indeed, able to minimize genetic damage of human lymphocytes induced by UVB, indicating the possibility of their usage against toxic hazards^3^. They have been studied to fight against foodborne pathogens likewise^4^. In terms of green synthesis of NPs, the approach is very eco-friendly as it consumes renewable waste materials to supplement the process^5^. The methods are simple and the NPs achieved are crystalline in a good degree^6,7^. The surface area of bio-synthesized α-Fe_2_O_3_ NPs were four times higher compared to the ones obtained by traditional methods and exhibited double reactivity^8^. Also, the green synthesis of metal and metalloid NPs is of interest due to their applications in removing potentially toxic elements from industrial effluents and recovering metals and metalloids from leachates and wastes of ore refining plants^9,10^.

Sulfur cell cycle is often used to produce green sulfide-metal NPs. Accordingly, a sulfate salt of the desired metal or metalloid is placed in contact with the living cells. First, sulfate salt enters the cell, and in the intracellular sulfur cycle; subsequently, it is converted into sulfite by intracellular enzymes, followed by being reduced to sulfide with the sulfite reductase enzyme. Therefore, obtaining metallic or metalloid sulfide NPs requires direct salt contact with living cells^11^. The production of NPs with supernatant can be an important step in the industrialization of the green production of sulfide NPs. As a result, considering the problems of NP extraction, a way to shorten the sulfate reduction path in a way the process does not require direct contact with cells and favors the extracellular production of NPs using the supernatant is very valuable. The importance may increase when we come to the green production of metalloid sulfide NPs, such as selenium sulfide, for which there are not many reports^12,13^.

Even if metal-sulfide NPs are produced outside the cell^14^, due to the dependence of sulfate reduction process on cell internal enzymes, it is impossible to use cultured cell supernatant to produce sulfide NPs, unless sulfate reducing enzymes were previously produced and released into the supernatant or sulfite was used as a substrate instead of sulfate. We applied both strategies, first of which, we added sulfate to the primary culture medium of the microorganism, one step before separating the supernatant, and in the second strategy, instead of sulfate substrate, we used sulfite substrate and thus shortened the sulfur reduction process. The sulfite reductase enzyme present in the supernatant was able to reduce the sulfite substrate.

**Figure 1:**
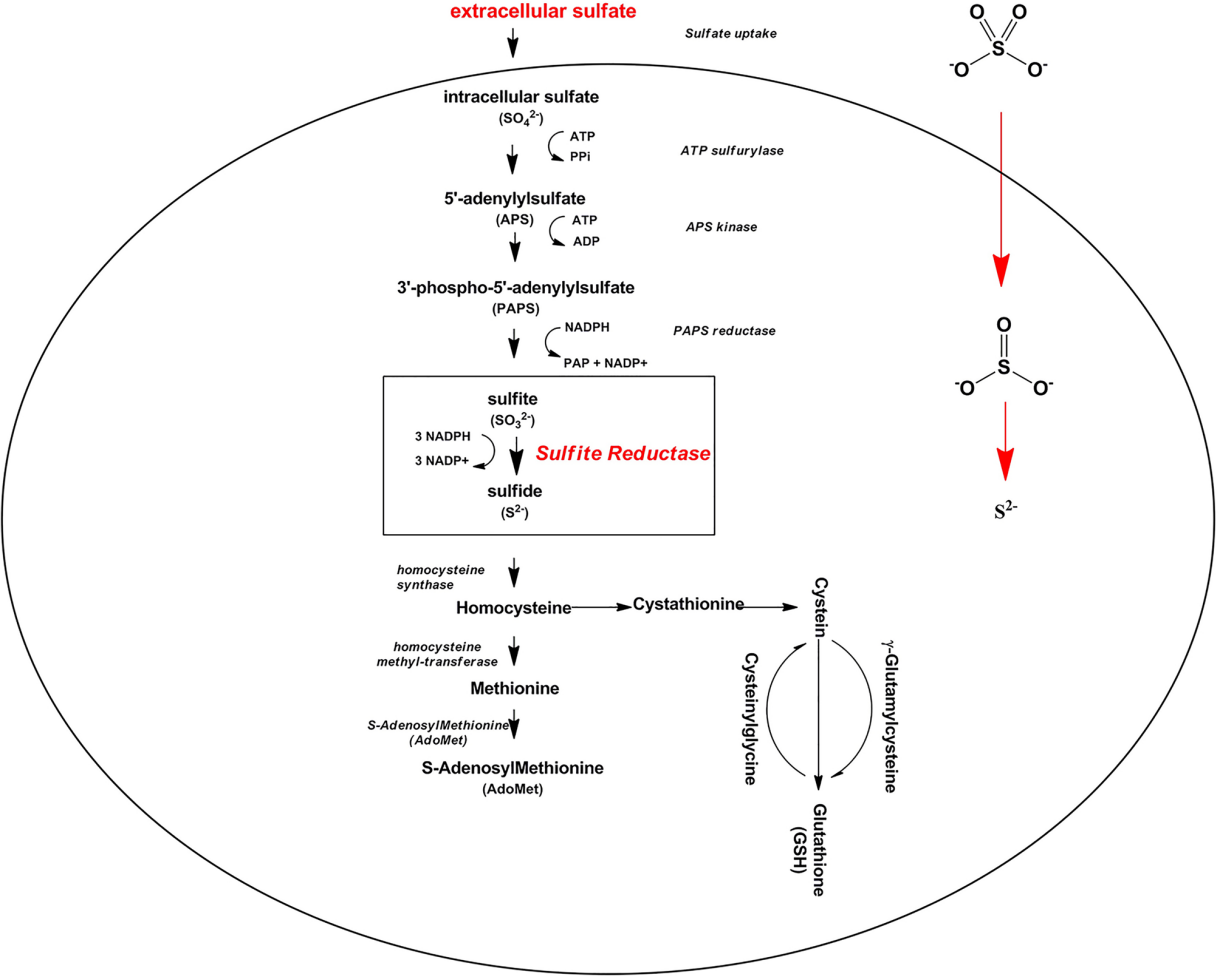
Sulfur metabolism pathway in *S. cerevisiae*. Sulfite reductase is a key enzyme for sulfide production in the cell. The primary substrate may be sulfate or sulfite.

We have previously obtained intracellular selenium sulfide NPs using *S. cerevisiae* and sodium selenosulfate (S_1_) as well as a combination of selenous acid and sodium sulfite salts (S_2_)^12^. Extracting NPs from yeast cells and their purification was a complicated and time-consuming process. Thus, we decided to explore the possibility of their extracellular production as mentioned above. Figure 1 depicts sulfur metabolism pathway in *S. cerevisiae*. Based on this hypothesis, we first performed four experimental models of extracellular selenium sulfide NPs bio-synthesis, described in Table 1. After determining which models were capable of extracellular synthesis of selenium sulfide NPs, we characterized the obtained NPs in terms of physiochemical characteristics.

**Table 1:** Detailed features of the models experimented in this study.

Sample	Selenium salts	Culture medium of *S. cerevisiae*	Ammonium sulfate added
S1−	Sodium selenosulfate	GYP*	No
S1 +	Sodium selenosulfate	GYP	Yes
S2−	Selenous acid/sodium sulfite (50/50)	GYP	No
S2 +	Selenous acid/sodium sulfite (50/50)	GYP	Yes

*GYP: glucose (2%)—yeast extract (1%)—peptone (2%) broth.

## Experimental details

### Materials and fungal strain

Peptone and yeast extract were purchased from Micromedia Trading House Co., Torbágyi út, Hungary. Sodium selenosulfate solution, ammonium sulfate, selenous acid and sodium dodecyl sulfate (SDS) were all obtained from Millipore Sigma Chemical Co., Milwaukee, USA and used with no further purification. Distilled water was prepared in-house by reverse osmosis technique using a Millipore S.A.S. France, Molsheim whose output water conductivity was 0.18 μs. *S. cerevisiae* (Persian Type Culture Collection 5052) was purchased from Iranian Research Organization for Science and Technology, Tehran, Iran.

### Biosynthesis of selenium sulfide NPs

*Saccharomyces cerevisiae* yeasts with a density of 10^6^ cells mL^−1^ were inoculated in 50 mL of glucose (2%)—yeast extract (1%)—peptone (2%) broth (GYP) medium and incubated at 35 °C with reciprocal shaking at 180 rpm using a shaking incubator (LabTech LSI-3016 R model, DAIHAN LABTECH CO. LTD., Republic of Korea). Ammonium sulfate was added to two series of the culture media to yield 1 mM final solution concentration.

After 24 h, cell suspensions were centrifuged at 3000 rpm for 10 min. The supernatant was passed through 0.22 μm JET BIOFIL® mixed cellulose ester (MCE) disk filters (Jet Bio-Filtration Co., Ltd., Guangzhou, China). Sodium selenosulfate solution (S_1_, 1 mM) and sodium sulfite/selenous acid (S_2_; 50/50 w/w, 1 mM) were added to the filtrate. The tests included a control containing 1 mM ammonium sulfate salt during cultivation of *S. cerevisiae* cells but without S_1_ or S_2_ salts. The necessary recurring abbreviations in this article are described in Table 1.

NPs were separated from the media by centrifugation using an ALLEGRA-64R centrifuge (Beckman Coulter Life Sciences Co., Indianapolis , USA) operating at 15,000 × g for 20 min followed by washing with SDS (1%) solution. The sediment was accordingly washed three times using deionized water at 42,000 × *g* for 1 hour.

### Characterization of NPs

Transmission electron microscopy (TEM) micrographs and selected area electron diffraction (SAED) patterns were performed on dried drops of NPs suspensions on carbon grids using a Tecnai F20 transmission electron microscope (Field Electron and Ion (FEI) Company, Hillsboro, USA) operating at an accelerating voltage of 50.0 kV and supported by an EDXA micro-analysis system. Additionally, an EDX micro-analysis system coupled to a MIRA3 scanning electron microscope (SEM) system (TESCAN, Brno, Czech Republic) and operating at an accelerating voltage of 15.0 kV was used for the purpose of elemental analysis of the specimens. Regarding SEM analysis, the isolated NP suspensions, dried on a glass or carbon substrate, were sputter coated by gold before SEM observations. Mean particle size and particle size distribution of at least 50 NPs were determined by image analysis using an image processing and analysis software, *i.e.* UTHSCSA Image Tool (Ver3.0.100), freely available from University of Texas Health Science Center San Antonio, Texas, USA.

XRD measurements were performed for the 2θ range of 3 to 80 degrees with a step of 0.0260 degree at a current of 40 mA using a Cu K-β (1.392 Å), K-α_1_ (1.540 Å) and K-α_2_ (1.544 Å) radiations and a voltage of 40 kV using a PANalytical X’Pert PRO MPD instrument. FTIR spectra were also obtained at a spectral resolution of 1 cm^−1^ using a Spectrum Two (PerkinElmer, USA) instrument operating at a wavelength range of 4000 to 400 cm^−1^. Samples were then prepared by mixing and compressing of NPs with KBr pellets. Mass spectrometry analysis was accordingly performed on an 5975C VL MSD instrument (Agilent Technologies, USA) equipped with a Triple-Axis Detector instrument operating from initial temperature of 50 °C with a rate of 70 °C.min^−1^ of increasing temperature up to 350 °C final temperature. Mass spectrometry data were analyzed using mMass software (Ver. 5.5.0) developed by Martin Strohalm in laboratory of molecular structure characterization of Institute of Microbiology, Prague, Czech Republic.

## Results and discussion

### Biosynthesis of Selenium sulfide NPs

**Figure 2:**
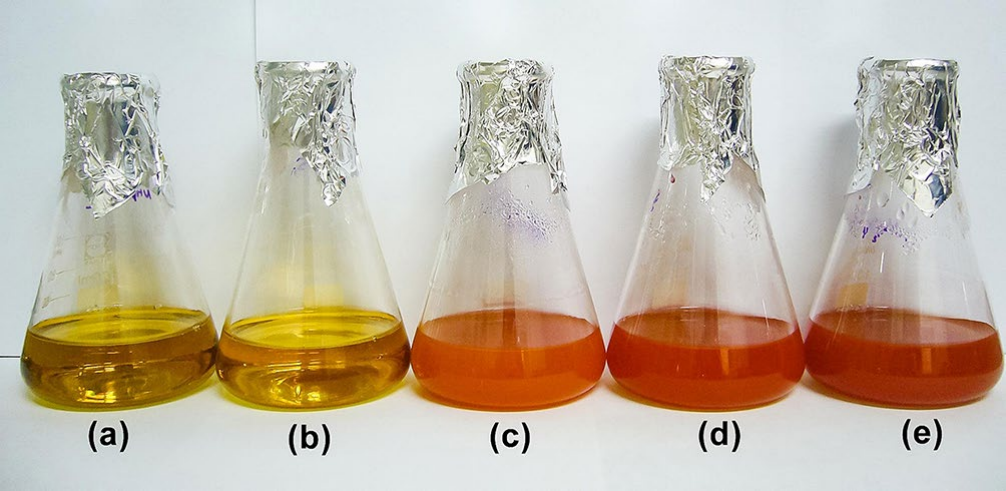
Extracellular production of selenium sulfide nanoparticles. The control lacked S_1_ and S_2_ salts but contained 1 mM ammonium sulfate during cultivation of *S. cerevisiae* cells (**a**). *S. cerevisiae* culture in S_1_- (**b**). *S. cerevisiae* culture in S_1_ +  (**c**). *S. cerevisiae* culture in S_2_− (**d**). *S. cerevisiae* culture in S_2_ +  (**e**). S_1_: Sodium selenosulfate; S_2_: Selenous acid/sodium sulfite.

**Figure 3:**
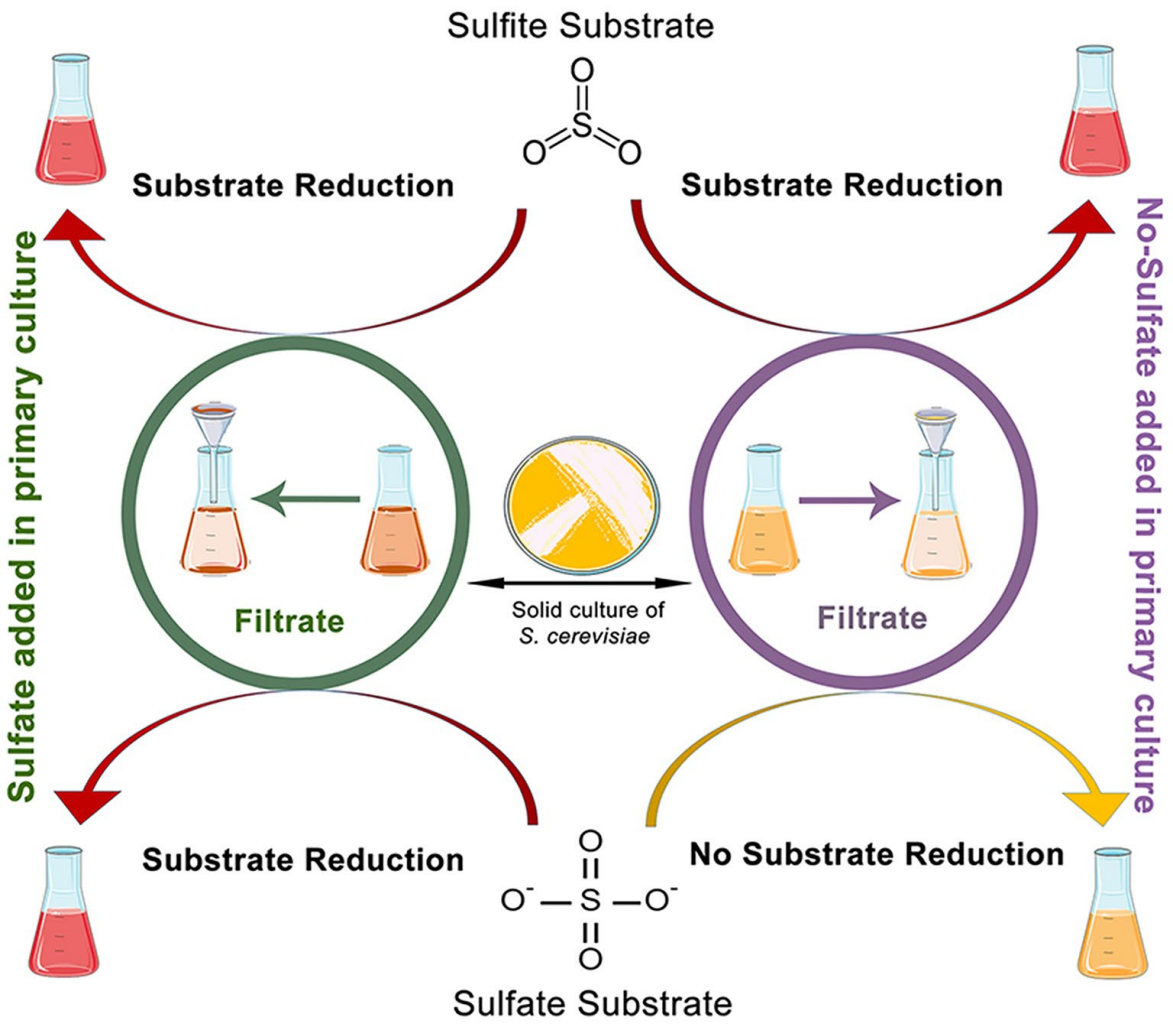
Visual description of the four strategies used for the extracellular synthesis of selenium sulfide nanoparticles and the results obtained.

**Figure 4:**
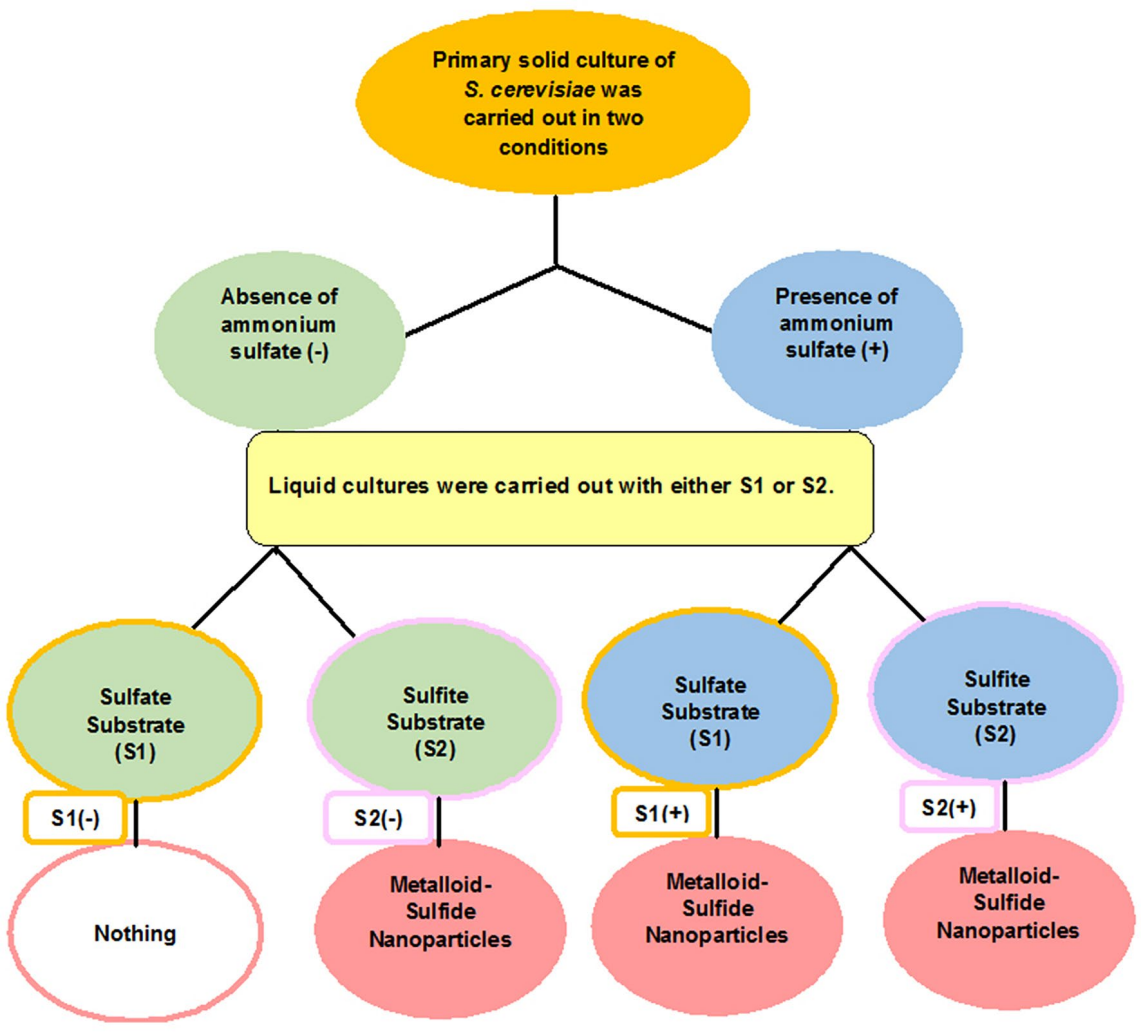
Descriptive diagram of the approaches for extracellular synthesis of metalloid sulfide in this study.

S_1_ (sodium selenosulfate) when cells had grown in the presence (S_1_ +) of ammonium sulfate beforehand, and S_2_ (selenous acid/sodium sulfite), while, cells were in the either presence (S_2_ +) or absence (S_2_−) of ammonium sulfate were all able to produce NPs (Fig. 2). The enzyme sulfite reductase needs six electrons to reduce sulfite to sulfide (S^2−^)^15^. The source of sulfur in S_2_ was sulfite, whereas, for the S_1_ this was sulfate. Sulfate, therefore, needed a further reduction step to become sulfite. While the microorganism itself was used instead of the supernatant in a previous research^12^ intracellular biosynthesis of the NPs occurred through the activity of sulfurylase enzyme located in the membrane of the microorganism reducing sulfate to sulfite, the sulfite reductase was then converted the sulfite to sulfide^16^. Therefore, the reaction in the S_1_ + suggests that exposure of the cells to ammonium sulfate might have caused the release of the enzyme sulfurylase in addition to sulfite reductase (Fig. 1). In the case of S_2_, since, the precursor was sulfite, it was reduced in the both cases (S_2_− and S_2_ +) due to sulfite reductase enzymatic activity. A summary of what happened is depicted in Figs. 3 and 4 provides supporting details of above information to clarify the main idea. Ahmad et al.^17^ previously reported on the determinant role of the reaction conditions on the extra and intracellular biosynthesis of gold NPs by a *Trichotheocium* species. They showed that the NPs were produced extracellularly when the flask of the biomass was kept in stationary conditions. Whereas, the intracellular NPs were obtained when the flask was shaken in a rate of 200 rpm. It seems under shaking conditions; gold ions were transported into interior of the fungal cell. However, they observed that no protein was released into the media when they were kept in shaking conditions^17^. In addition, reduction of selenite was mediated by *Rhodospirillum rubrum*, a phototrophic bacterium. The obtained selenium particles were observed both inside and outside the microorganism. It was not clear whether the particles were originated from intracellular sources or were formed in the extracellular space. According to findings reported by Kessi et al.^18^, some molecules existing in the stationary phase of the bacteria were responsible for reduction reaction. They also found that cell division process was affected by selenite^18^.

### Characteristics of NPs

The requirements for salts reduction, *e.g.* the proton donor, sulfur carriers as well as the enzyme were all provided by the cells supernatants. The research circumstances, including the microorganism, culture media, pH, temperature and speed of shaking were indeed, the same among different treatments excluding the presence or absence of the ammonium sulfate precursor in the initial medium. The resulting NPs were characterized by SEM, TEM, SAED, EDX, FTIR, mass spectrometry and XRD techniques.

**Figure 5:**
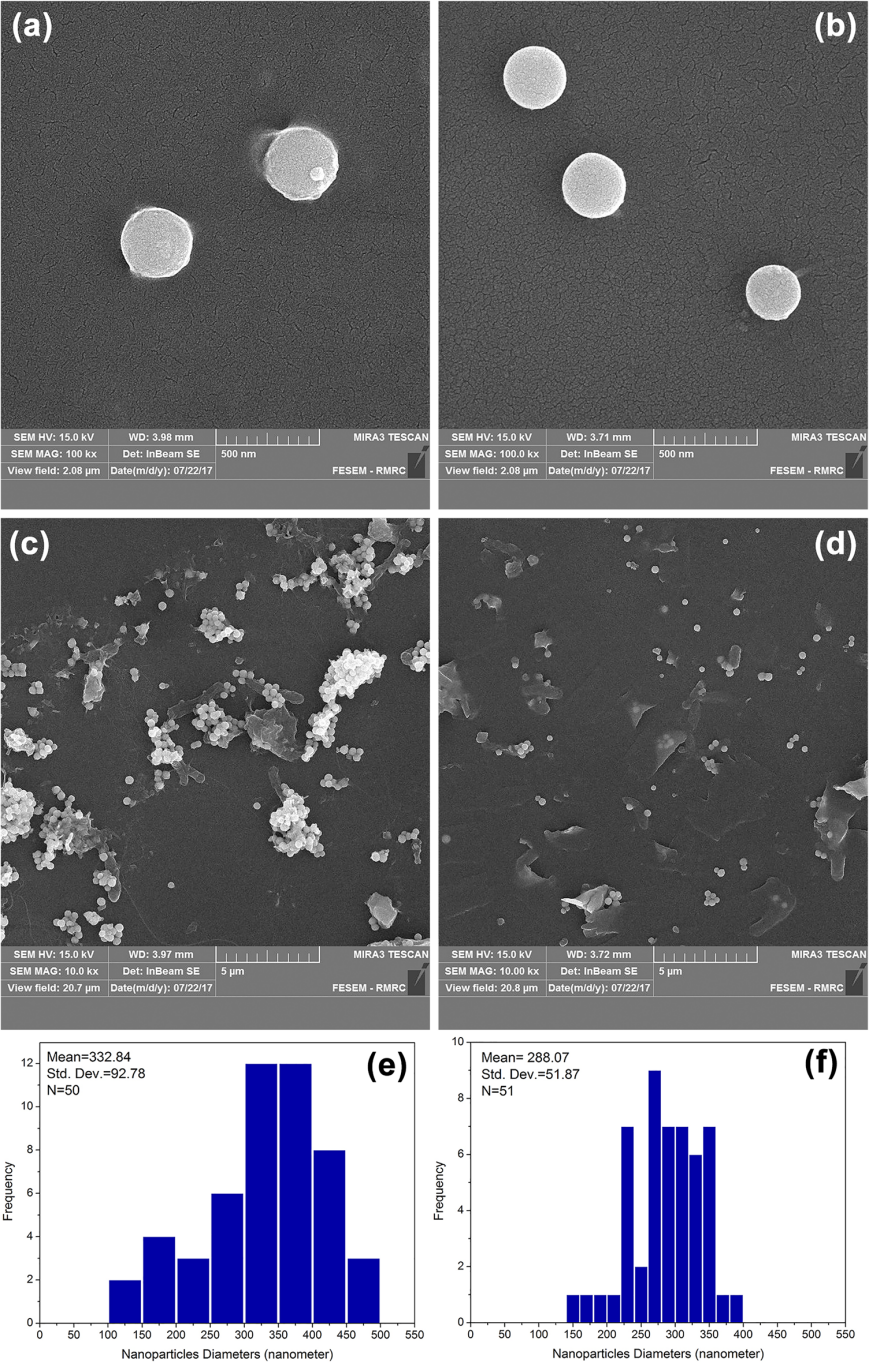
SEM photomicrographs of nanoparticles in different magnification ratio and their particle size distribution histograms in S_2_− (**a**, **c** and **e**) and S_2_ + (**b**, **d** and **f**).

The SEM micrographs visualized the morphology of selenium sulfide NPs bio-synthesized by the supernatant of *S. cerevisiae* culture. According to SEM images (Fig. 5), both S_2_+ and S_2_− NPs demonstrated spherical structures with average particle size of 288 nm for S_2_+ and 332 nm for S_2_−. In S_2_− samples, where ammonium sulfate was not used in the primary culture of *S. cerevisiae*, the average size of nanoparticles was approximately 333 nm. While, the size of nanoparticles was between 100 and 500 nm and the most numbers were between 300 and 400 nm. In S2+ samples, where ammonium sulfate was used in the primary culture of *S. cerevisiae*, the range of nanoparticle size distribution was found to be narrower, and the most number of nanoparticles were in the range of 260 to 280 nm. The morphology of the nanoparticles was shown similar in terms of geometric shape and both were spherical.

In a previous study by the same authors, obtaining intracellular selenium sulfide NPs, S_2_− was about 153 nm in SEM micrographs^12^. Vogel et al.^19^ managed to biosynthesize selenium sulfur NPs from selenite using bacterium *Azospirillum brasilense*. The extracellular NPs of Se_8-n_S_n_ were stable having a spherical morphology with a mean particle size of around 400 nm. They, accordingly, noticed that sulfur was involved in the selenium NPs formation. However, in low concentration of sulfate (≤ 300 mg L^−1^) the pure selenium NPs were detected according to EDX analysis. Unlike the previous one, a high concentration of sulfate (850 mg L^−1^) resulted in development of sulfur-selenium structures. Prasad et al.^3^ successfully synthesized selenium NPs using aqueous leaf extract of lemon plant. In a study by Zare et al.^20^, *Aspergillus terreus* was isolated from soil and used for intracellular synthesis of selenium NPs (47 nm) within 60 min.

**Figure 6:**
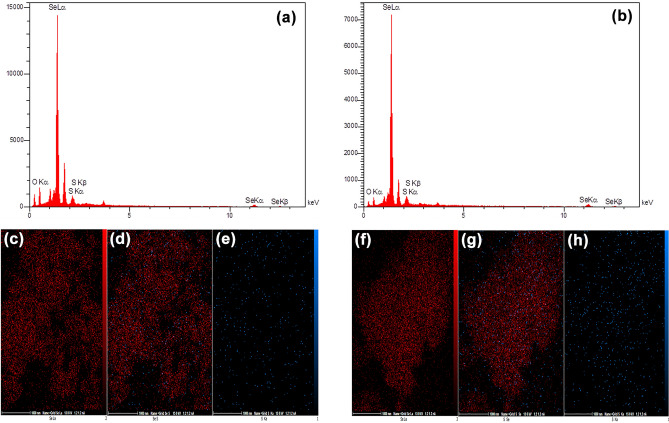
EDX diffractogram of S_2_− (a) with detailed pictures of the mapping of selenium (**c**), the combination of selenium and sulfur (**d**) and sulfur (**e**). EDX diffractogram of S_2_ + (b) with detailed pictures of the mapping of selenium (**f**), the combination of selenium and sulfur (**g**) and sulfur (**h**).

**Figure 7:**
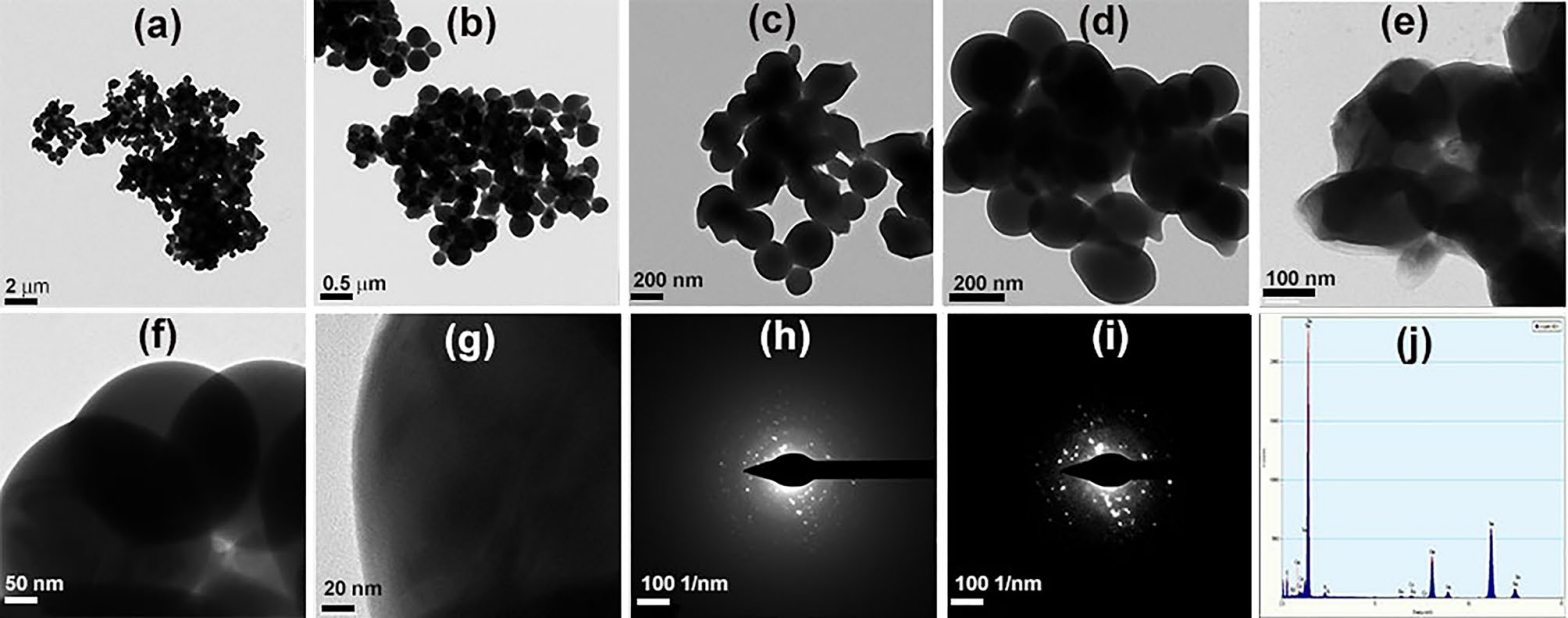
Detailed features of the morphology, crystallization and the elemental contents of S_2_− NPs are shown: (**a**–**g**) TEM micrographs recorded at different magnifications ratio, (**h** and **i**) SAED patterns of S_2_− NPs and (**j**) EDX spectrum of S_2_− NPs.

**Figure 8:**
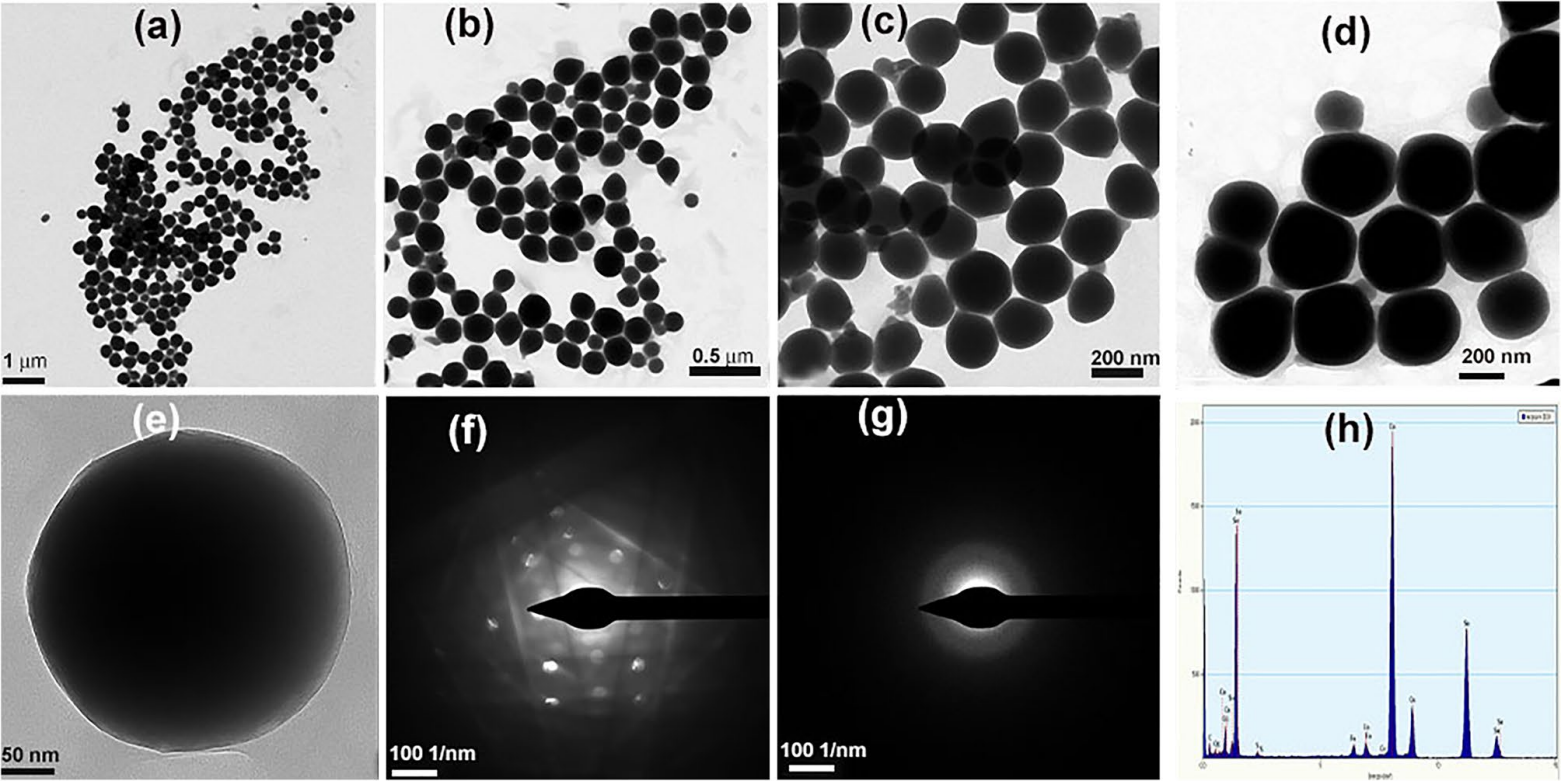
Detailed features of the morphology, crystallization and the elemental contents of S_2_ + NPs are shown: (**a**–**e**) TEM micrographs of recorded at different magnifications, (**f** and **g**) SAED patterns of S_2_− NPs and (**h**) EDX spectrum of S_2_ + NPs.

Furthermore, EDX and elemental mapping were conducted on the particles using FESEM technique (Fig. 6) confirming the presence of the selenium and sulfur elements in the NPs formed in the S_2_− and S_2_ + treatments. The proportion of selenium to sulfur for S_2_− and S_2_ + were 24.50 and 23.31, respectively, which can be regarded the same with no meaningful difference (Fig. 6). In TEM micrographs, more precise vision of morphology and particle size distribution of the selenium sulfide NPs were provided (Figs. 7 and 8). TEM microscopy indicated that average size of 268 and 305 nm for S_2_ + and S_2_−, respectively (Figs. 7 and 8). Additionally, it was confirmed that the spherical NPs consisted of selenium and sulfur. The individual NPs were demonstrated among and inside a number of aggregates, suggesting a protein capping agent stabilized the NPs. SAED patterns are shown in Figs. 7 and 8. Based on distinct bright rings in the image h in S_2_−, and f in the S_2_ + , the preferential orientation of nanocrystals in these particles was confirmed, too. Only the image i in the S_2_− seems to be a diffraction of an amorphous particle. Hence, it was suggested that the particles were selenium sulfide; while, the intensity of the sulfur peak is very low, implying that there may be a modified version of selenium sulfide, which was subsequently confirmed by the mass spectrometry results. Nanoparticles obtained from S_2_ + , which ammonium sulfate was used in their initial culture of *S. cerevisiae* cells, were crystallized better.

**Figure 9:**
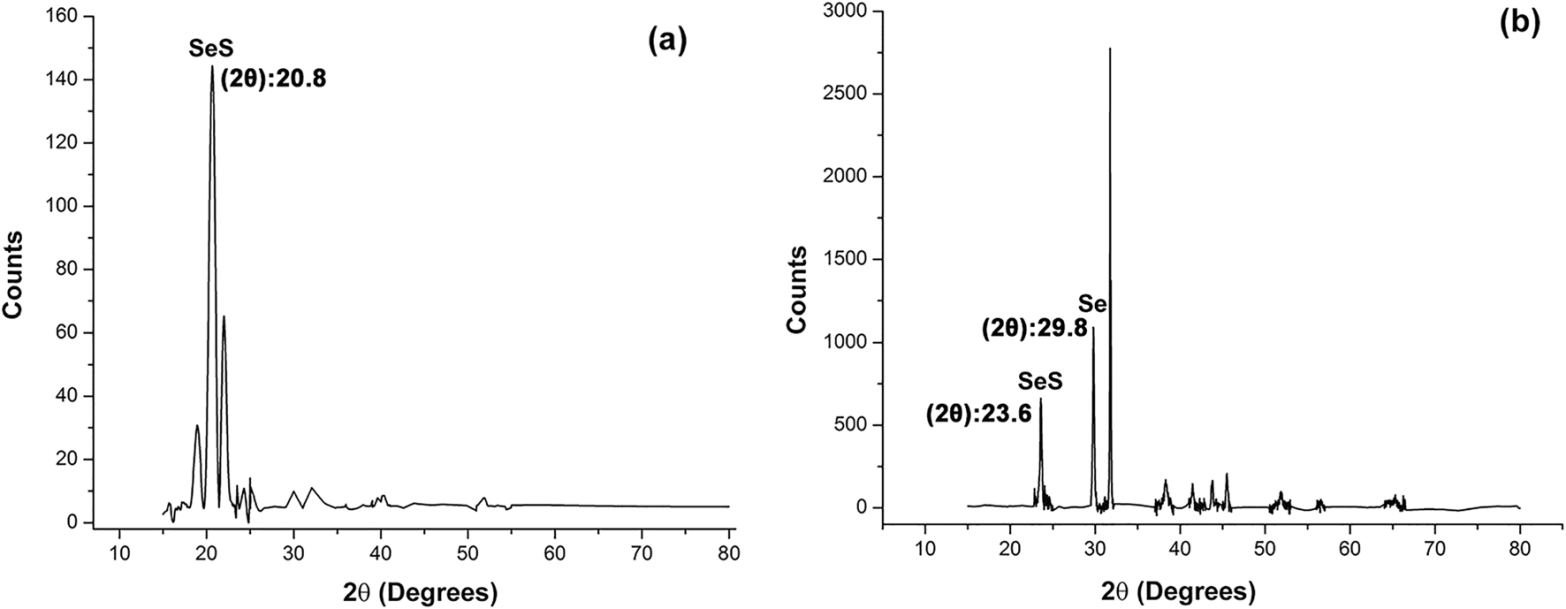
XRD diffraction patterns of bio-fabricated selenium sulfide nanoparticles: S_2_− (**a**) and S_2_ + (**b**).

**Figure 10:**
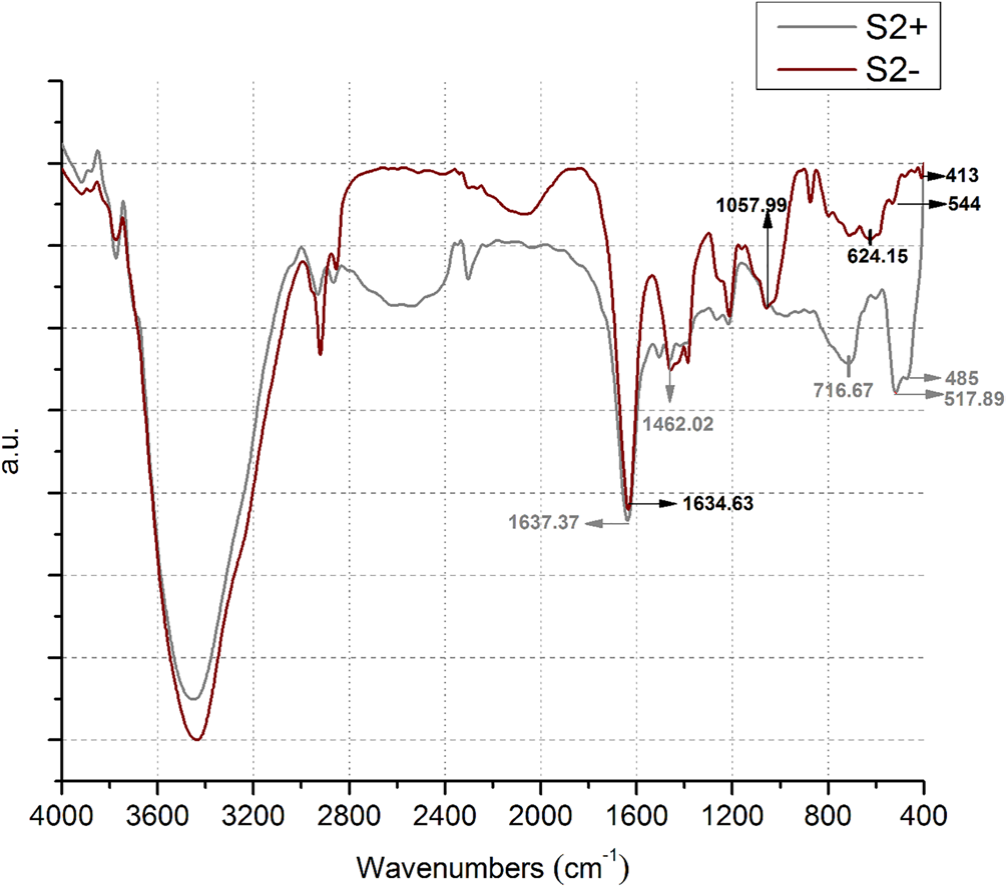
FTIR pattern of S_2_− (in the absence of ammonium sulfate in fungal cultures) and S_2_ + (by adding ammonium sulfate to fungal culture) bio-nanoparticles.

In order to find out the exact proportion of selenium or sulfur in the NPs, mass spectrometry was carried out for S_2_ + and S_2_−. Therefore, this proposed a high chance of the existence of Se, Se_2_, Se_3_S_5_/ Se_5_, Se_6_ and Se_4_ species for S_2_− and Se_2_S_2_, SeS_1.1_, Se_2_, Se, SeS_5_, SeS_3_, Se_3_S_5_/Se_5_, Se_3_/SeS_5_, Se_6_, Se_4_/SeS_7_, Se_2.57_S_5.43_/ Se_2_S_2_ and Se_4_S/Se_2_S_6_ molecules for S_2_ + treatments. Existence of selenium in a variety of stoichiometric ratios with numerous chemical properties is due to the various potential oxidation states (− 2, 0, + 2, + 4 and + 6) possible. In almost all inorganic as well as organic compounds, sulfur can be replaced with selenium^21^. In the XRD pattern of S_2_ + NPs (Fig. 9), peaks possessing the strongest Bragg’s reflections showed the closest match with SeS and Se according to standard JCPDS cards 00-002-0320 and 00-0042-1425, respectively and that of S_2_− NPs revealed a peak with closest match with SeS in relation to standard JCPDS card 00-002-0320. XRD data, whereas, coincided with SAED pattern of S_2_− and S_2_ + . The crystallinity of selenium-sulfur composition is influenced by the ratio of sulfur. A 50% proportion of sulfur entirely changes the diffraction pattern of selenium, resulting in the appearance of new phases and peaks^22^. Based on Scherrer equation, the crystal size for S_2_− sample was 53.62 nm and for the S_2_ + sample, was 42.40 nm. The size of the crystals, as well as the average size of the particles which were 268 and 305 nm in TEM, was larger in S_2_− sample than that of S_2_ + . In FTIR spectra, carried out after purification of S_2_− and S_2_ +  (Fig. 10), the maximum absorption of sulfide in both S_2_− and S_2_ + was among 400 and 500 cm^−1^ and more prominent peaks were found between 450 and 500 cm^−1^, in S_2_ + which is in consistency with^23^. Primary (1090–1020 and 1650–1580 cm^−1^) as well as secondary (1580–1490 cm^−1^) amine bands^24^ were also present in FTIR spectra confirming the protein corona has been formed around NPs.

There are, though, more examples of extracellular biosynthesis of NPs. Othman et al., (2017) successfully biosynthesized silver NPs extracellularly mediated by *Trichoderma viride*. They used the filtrate of the fungus medium, transferred via Whatman filter paper No 1 to obtain the silver NPs^25^. Using the cell-free supernatant of a Gram-positive bacteria, *Serratia nematodiphila*, extracellular zinc sulfide was also produced^11^. When extracellular synthesis of NPs is elaborated, two states may emerge including synthesis in the presence of microorganisms and synthesis with cell-free filtrate/supernatant in the absence of microorganisms.

Concerning the first case, it is not often known that the NPs were first made inside the cell and then transferred out, or vice versa. In such a case, the purification of NPs, such as their intracellular production, is seemingly difficult. Tugarova et al. (2020) developed a method to synthesize extracellular selenium NPs using two strains of a rhizobacterium, *i.e. Azospirillum brasilense*. A live biomass of *A. brasilense* in its logarithmic phase were also used to reduce selenite (${\text{SeO}}_{3}^{2 - }$) to SeNPs in a nutrient-free saline solution. A combination of centrifugation and filtering was accordingly utilized to purify SeNPs^26^. In this case, a cell-free culture medium was used to synthesize NPs aiming to obtain NPs in a separate state from microorganisms in order to be easily purified. Further, it was known that *S. cerevisiae* produces the enzyme(s) reducing selenium salts^12,27^, the question was that whether *S. cerevisiae* produces the enzyme(s) extracellularly, or secrets them out of the cell after production, and if not, can exposing them to a salt such as ammonium sulfate, during primary culture, stimulate the cells to produce the enzyme(s)? Silva et al.^25^ performed extracellular synthesis of highly stable silver NPs using a fungus named *Duddingtonia flagrans*. The authors used cell-free filtrate of the fungus and AgNO_3_. However, it was found that the type of medium of the biomass influenced the amount of total proteins in the fungal filtrate. It is therefore, assumed that the fungus was stimulated to produce some enzymes based on the components of the medium. It appears that enriching the medium with thick shells as a natural source of chitin resulted in the production of chitinase. In our experience, the presence of ammonium sulfate in the primary culture medium (before cell/supernatant filtration) for the production of S_2_ NPs (using sulfite substrate) was not essential. Whereas, this was not the case with S_1_ (using sulfate substrate), as the presence of ammonium sulfate in the primary culture medium was critical for the production of NPs with its cell-free supernatant. As previously mentioned, precursor S_1_ was a sulfate that required membrane sulfurylase enzymes to be reduced to sulfite followed by being converted to sulfide by the enzyme sulfite reductase. The difference in cell function against these two precursors suggests that more enzymes were required to reduce S_1_ into selenium sulfide NPs. The enzymes that were not produced extracellularly except in the presence of stimulant salts such as ammonium sulfate, were able to reduce S_1_. As shown before^12^, intracellular production of these NPs was possible with both precursors and under similar conditions. It seems that for extracellular production of selenium sulfide NPs with *S. cerevisiae*, it is either possible to use sulfite salts or in the primary culture, the fungus is exposed to a sulfate salt to produce the enzyme and release it. In addition, our results showed that sulfite reductase is naturally released outside the cell; whereas, membrane enzymes involved in sulfate reduction are released outside the cell only after encountering sulfate.

## Conclusions

In this study, selenium sulfide NPs were synthesized extracellularly using a yeast factory, *S. cerevisiae*. Between two sulfate (S_1_) and sulfite (S_2_) substrates, sulfite (S_2_) is preferable. Since the sulfite substrate provided a greater possibility for the synthesis of metalloid sulfide, both in the presence and absence of ammonium sulfate. The size of nanoparticles in the samples obtained from sulfite precursor, which ammonium sulfate was used in the initial culture of *S. cerevisiae* (S_2_ +), was between 200 and 300 nm. This particle size is, therefore, very suitable for drug delivery. Also, in addition to the benefits of green methods in the synthesis of these NPs including usage of non-toxic and nature-friendly materials, conservation of resources and easy access to raw materials, it has further advantage of no need for laboring and time-consuming down-stream process of NPs purification from producing microorganism. Furthermore, the separated NPs not only have better purity than those produced intracellularly but also their industrial production will be facilitated in large-scale quantities.

## Data Availability

The datasets used in the current study are not publicly available. For non-commercial use, other researchers can obtain them upon reasonable request from MRA (mrab442@yahoo.com) and MI (M.Imani@ippi.ac.ir).
